# Impact of SAA versus CRP in evaluation of acute-phase response in familial Mediterranean fever: A prospective study

**DOI:** 10.1097/MD.0000000000050309

**Published:** 2026-08-21

**Authors:** Oguzhan Selvi, Bugra Han Egeli, Kerem Parlar, Elif Kilic Konte, Mustafa Akker, Mert Oztas, Sezgin Sahin, Amra Adrovic, Kenan Barut, Ibrahim Murat Bolayirli, Osman Simsek, Ozgur Kasapcopur, Huri Ozdogan, Serdal Ugurlu

**Affiliations:** a Department of Internal Medicine, Cerrahpasa Medical Faculty, University of Istanbul-Cerrahpasa, Istanbul, Turkey; b Department of Pediatrics, Cerrahpasa Medical Faculty, University of Istanbul-Cerrahpasa, Istanbul, Turkey; c Department of Intensive Care, Cerrahpasa Medical Faculty, University of Istanbul-Cerrahpasa, Istanbul, Turkey; d Department of Internal Medicine, Division of Rheumatology, Cerrahpasa Medical Faculty, University of Istanbul-Cerrahpasa, Istanbul, Turkey; e Department of Pediatrics, Division of Rheumatology, Cerrahpasa Medical Faculty, University of Istanbul-Cerrahpasa, Istanbul, Turkey; f Department of Biochemistry, Cerrahpasa Medical Faculty, University of Istanbul-Cerrahpasa, Istanbul, Turkey; g Department of General Surgery, Cerrahpasa Medical Faculty, University of Istanbul-Cerrahpasa, Istanbul, Turkey.

**Keywords:** acute-phase reactant, familial Mediterranean fever, serum amyloid A

## Abstract

To compare the sensitivity and prognostic value of SAA and C-reactive protein (CRP) during familial Mediterranean fever (FMF) attacks and attack-free periods. Patients with juvenile idiopathic arthritis (JIA), acute appendicitis, and sepsis were used as controls. In this prospective study, 42 patients with FMF, 30 with JIA, and 30 with acute appendicitis were included. Blood samples for CRP and SAA were collected at 2 different time points: first, during an attack or preoperatively for acute appendicitis cases, and second, after the subsidence of the attack or postoperatively. To evaluate the role of SAA and CRP in the follow-up of inflammation, 42 patients with sepsis were studied serially. Twenty healthy individuals were also tested as controls. In appendectomy, JIA, and FMF patients, the mean CRP and SAA levels returned to normal values after operation/attack treatment. In both the inflammatory and non-inflammatory phases, a positive correlation was detected between CRP and SAA levels (*P* < .001). In the sepsis group, mean SAA and CRP levels were 221.21 ± 95,05 and 402.2 ± 255.4 respectively. CRP and SAA levels were consecutively measured in 19 patients with sepsis, and a positive correlation was detected between these markers (*P* < 0.01). In healthy controls, the SAA and CRP levels were within the normal ranges. We report that SAA and CRP levels were correlated in various disease settings. We suggest that CRP measurements are adequate for evaluating acute inflammatory conditions, and follow-up with SAA does not contribute to the early recognition of subclinical inflammation.

## 1. Introduction

Familial Mediterranean fever (FMF) is the most common hereditary autoinflammatory disease, characterized by fever and serositis attacks.^[1]^ FMF, considered an autosomal recessive disease, is associated with mutations in the Mediterranean fever gene. However, in approximately one-third of FMF cases, even in high-risk populations, the results of genetic testing are not diagnostic. Currently, the diagnosis of FMF remains clinically important.^[2]^ The most common symptoms observed during FMF attacks are fever, abdominal pain, chest pain, arthritis, and erysipelas-like erythema (ERE). Inflammatory attacks are accompanied by an elevated acute-phase response, which decreases as the episode subsides. Persistence of an acute-phase response during attack-free intervals is a sign of subclinical inflammation and, thus, the ongoing risk of amyloid A amyloidosis, the most fatal complication of FMF.^[3]^ Life-long prophylactic colchicine is the treatment of choice that prevents the recurrence of attacks and the development of amyloidosis in the majority of compliant patients. Subclinical inflammation requires regular laboratory follow-up to evaluate the response to colchicine treatment.^[4,5]^ Anti-interleukin-1 agents, anakinra and canakinumab, are effective treatment options for colchine-resistant or colchine-intolerant cases.^[6]^

The C-reactive protein (CRP), serum amyloid A (SAA), erythrocyte sedimentation rate, fibrinogen, and leukocyte counts provide valuable information. Among these, SAA and CRP have been established in the literature and clinical environments. These 2 markers are the most responsive and dynamic; however, their differences have not been adequately investigated. As CRP is readily available in routine clinical practice, the superiority of SAA over CRP should be certain to replace CRP. European Alliance of Associations for Rheumatology (EULAR) guidelines from 2015 also proposed the use of SAA for the regular follow-up of patients.^[7]^ In amyloid A amyloidosis-complicated FMF patients, sustained elevation of SAA is almost a prerequisite.^[8]^ However, SAA and CRP levels are correlated with various rheumatic inflammatory conditions.^[9]^ The same correlation also appears to exist for several viral and bacterial infections.^[10]^

Therefore, the main objective of this study was to understand and compare the sensitivity and prognostic value of SAA and CRP during FMF attacks and attack-free periods.

## 2. Materials and methods

The patients included in this study were FMF patients who were followed up at our rheumatology outpatient clinic. Multiple positive controls were used to test the validity of the results. The positive control group included patients diagnosed with sepsis, acute appendicitis, or juvenile idiopathic arthritis (JIA). The healthy control group consisted of healthy participants. The study included 42 patients with FMF, 30 with acute appendicitis, 42 with sepsis, 30 with JIA, and 20 healthy controls.

The study protocol was approved by the Istanbul University-Cerrahpasa, Cerrahpasa Medical Faculty Ethics Committee (Approval number: 83045809-604.01.12). Written informed consent was obtained from all the patients. This study was conducted in accordance with the principles of the Declaration of Helsinki.

Venous blood samples were drawn from all patients to evaluate the CRP and SAA levels after 10 to 12 hours of overnight fasting. For each patient, 2 blood samples were drawn: 1 during the inflammatory phase and the other after the resolution of inflammation, whether it was an FMF attack or an infection. The samples were centrifuged for 15 minutes at 1000 × g. Serum samples were separated, aliquoted, frozen, and stored at −20 ° C until SAA analysis. The serum CRP levels were measured on the same day. For these parameters, the intra- and inter-coefficient of variation were 5.1% and 8%, respectively. Serum CRP levels were measured using an immunoturbidimetric method in the Roche Modular System (Roche Diagnostics, Germany) and were expressed as mg/L. The upper limit of the reference range for CRP was 5 mg/L. SAA levels were measured using the nephelometric method with a Siemens Behring Nephelometer 2 system. The upper limit of the reference range for SAA was determined as 6.8 mg/L. The requisite for CRP and SAA to be considered elevated is for the levels to be at least 1.5 times higher than the upper normal limit.

In the statistical evaluation, the data are presented as mean ± standard deviation and percentage. Descriptive statistics were used to describe basic data features. Shapiro–Wilk normality test was used to evaluate the normality of the distribution of numeric variables. Student *t* test was used for the analysis of parametric data with normal distribution, while the Mann–Whitney *U* test was used for those without normal distribution. The Chi-square test was used to compare data in groups, and evaluation was performed using Fischer’s exact test. Statistical significance was set at *P* < .05. Pearson’s test was used to assess the correlation between the continuous data. Statistical Package for the Social Sciences (SPSS) for Windows version 14.0 software (SPSS Inc., Chicago) was used for the analysis.

## 3. Results

One-hundred-sixty-four patients were included in this study in 5 different groups. The demographic and clinical characteristics of the different groups are shown in Table 1. Mediterranean fever gene mutations of FMF patients tested with next-generation sequencing is shown in Table 2.

**Table 1:** Demographic and clinical characteristics of the different groups.

	FMF	Acute appendicitis	Sepsis	JIA	Healthy controls
	(n = 42)	(n = 30)	(N = 42)	(n = 30)	(n = 20)
Age, mean, ± standard deviation, yr	28.31 ± 11.75	30.5 ± 11.14	63.76 ± 15.17	13.05 ± 4.62	31.44 ± 10.41
Gender M/F	15/27	17/13	23/19	14/16	9/11
Disease duration, mean, ± standard deviation, yr	19.75 ± 10.54	N/A	N/A	7.4 ± 3.47	N/A
Delay in disease diagnosis, mean, ± standard deviation, yr	5.56 ± 9.08	N/A	N/A	0.55 ± 0.89	N/A

FMF = familial Mediterranean fever, JIA = juvenile idiopathic arthritis.

**Table 2:** MEFV gene mutations of the FMF patients.

MEFV gene mutation	n = 42
M694V homozygous	27 (64%)
M680I/M694V compound heterozygous	3 (7%)
M680I homozygous	2 (5%)
F479L/V726A compound heterozygous	3 (7%)
E148Q heterozygous	1 (2%)
R761H/R202Q compound heterozygous	1 (2%)
V726A heterozygous	1 (2%)
M694V/V726A compound heterozygous	1 (2%)
M680I/V726A compound heterozygous	1 (2%)
Unspecified	2 (5%)

FMF = familial Mediterranean fever, MEFV = Mediterranean fever gene.

Among 42 patients in the FMF group, 27 had peritonitis, 10 had arthritis, 1 had pericarditis, 1 had febrile myalgia, and 7 had both peritonitis and pleuritic attacks. One patient had a history of renal transplantation for FMF-related amyloidosis.

All the patients in the FMF group were treated with prophylactic colchicine. Nine patients were treated with additional biological therapies (6 with anakinra, 1 with adalimumab, and 2 with canakinumab). In 1 patient with arthritis and 1 patient with peritonitis/pleuritis, anakinra treatment was started after the blood was drawn; hence, the attack-free blood sample was under the effect of anakinra, unlike the sample during the attack. Two patients were treated with anakinra and 3 patients were treated with canakinumab due to colchicine-resistant serositis attacks. In all the patients, a second round of blood tests was performed during the attack-free period.

Patients with acute appendicitis had rebound tenderness during admission, were diagnosed with imaging, and were treated surgically. Serum samples were drawn before surgery and 4.28 ± 4.17 months after surgery. All patients were asymptomatic during the latter period. None of the patients had received any medical treatment.

The JIA group included patients who were followed up by pediatric rheumatology physicians at our institution. These patients had active joint complaints when the blood was drawn. Of the 30 patients, 12 had oligoarticular JIA, 13 had polyarticular JIA, 2 had systemic JIA, 1 had psoriatic JIA, 1 had juvenile spondyloarthritis, and 1 was unknown. Five patients received intra-articular steroid injections on the same day. Nineteen patients received methotrexate and oral steroid treatment, 5 received leflunomide and oral prednisolone treatment, 3 received methotrexate, oral prednisolone, and etanercept treatment, and 3 received leflunomide, tocilizumab, and oral prednisolone treatment. Two patients were not receiving any treatment prior to their complaints due to disease remission. The follow-up blood samples were drawn when the patients were in remission 8.16 ± 5.41 months later.

In the sepsis group, all 42 patients were followed up in our intensive care unit. All patients were treated with broad-spectrum antibiotics and 20 (47%) received inotropic circulatory support. The primary sources of infection were the lungs in 16 patients, the abdomen in 16 patients, the surgical site in 5 patients, the endocardium in 1 patient, and pressure ulcer in 1 patient. Three patients had opportunistic bloodstream infection.

**Figure 1.**
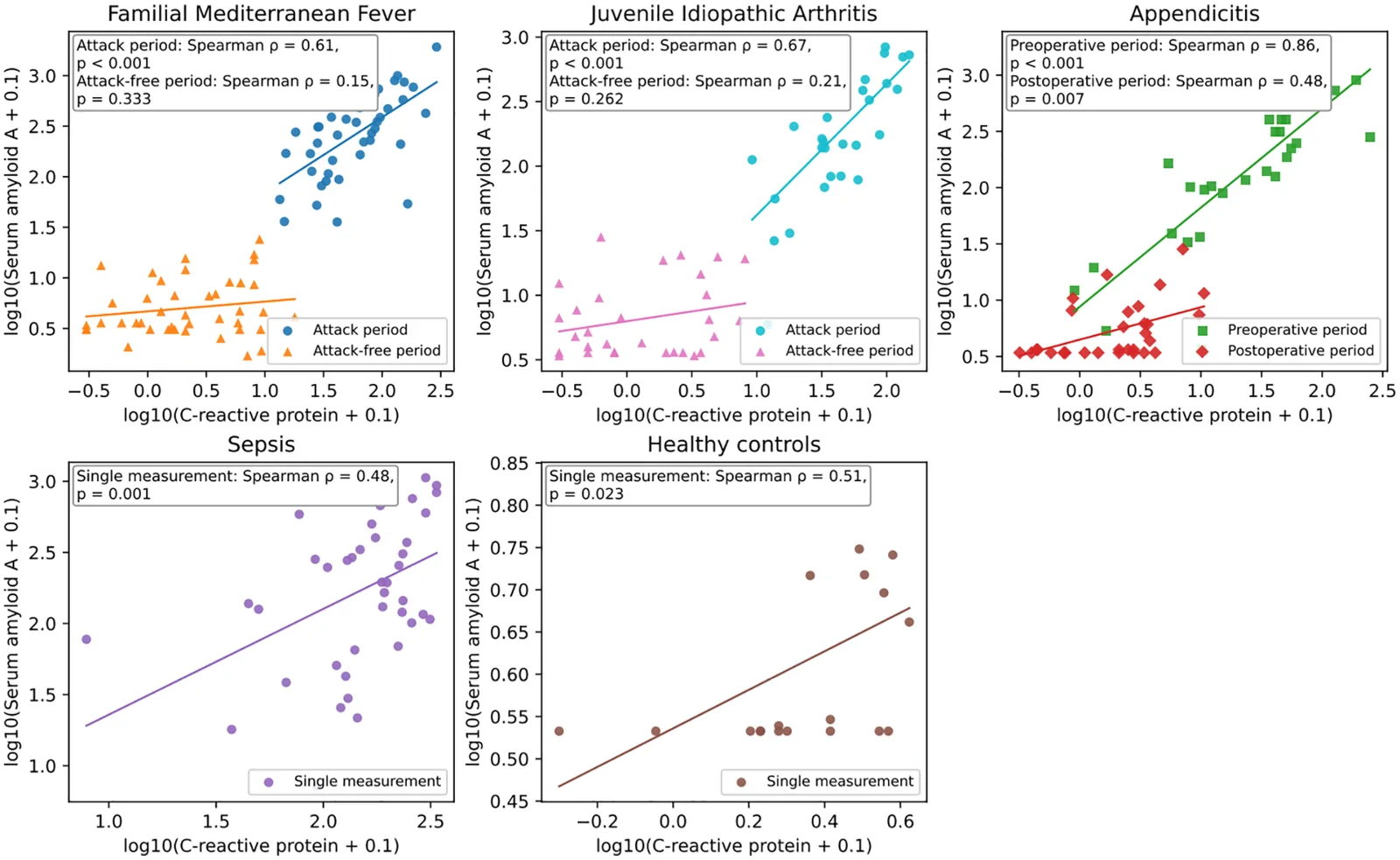
Correlation between serum amyloid A and C-reactive protein levels. Scatter plots showing the relationship between serum amyloid A (SAA) and C-reactive protein (CRP) levels in patients with familial Mediterranean fever (FMF), juvenile idiopathic arthritis (JIA), acute appendicitis, sepsis, and healthy controls. For FMF and JIA, measurements obtained during the attack period and the attack-free period are presented separately. For appendicitis, preoperative and postoperative measurements are shown. In the sepsis and healthy control groups, single measurements are displayed. Both SAA and CRP values are expressed as log10-transformed values [log10(SAA + 0.1) and log10(CRP + 0.1)]. Spearman correlation coefficients (ρ) and corresponding *P* values are indicated in each panel. CRP = C-reactive protein, FMF = familial Mediterranean fever, JIA = juvenile idiopathic arthritis, SAA = serum amyloid A.

The SAA and CRP levels of the patients during the attack and attack-free periods are shown in Table 3. In all groups, SAA and CRP levels were significantly higher during the attack period. In patients with FMF, acute appendicitis, and JIA, SAA, and CRP levels were found to be correlated with each other (FMF: *r* = 0.692, *P* < .01), acute appendicitis (*r* = 0.651, *P* < .01), and JIA (*r* = 0.719, *P* < .01; Fig. 1). In the sepsis group, CRP and SAA levels were correlated in 41 patients, with 82 measurements.

**Table 3:** SAA and CRP levels of the patients during attacks and attack-free periods.

	FMF (n = 42)	Acute appendicitis (n = 30)	Sepsis (n = 42)	JIA (n = 30)	Healthy controls (n = 20)
SAA during inflammatory phase (mg/L) attack/pre op	380.35 ± 355.1	307.05 ± 371.72	309.22 ± 309.9	274.36 ± 265.95	4.1 ± 1.2
SAA non-inflammatory phase (mg/L) attack-free/post op	6.2 ± 4.88	6.44 ± 5.39	N/A	7.38 ± 6.82	N/A
*P*	<.01	<.01	N/A	<.01	N/A
CRP during inflammatory phase (mg/L) attack/pre op	77.11 ± 62.55	49.02 ± 57	177.22 ± 85.33	53.89 ± 43.29	2.4 ± 1
CRP during non-inflammatory phase (mg/L) attack-free/post op	3.7 ± 3.76	2.69 ± 2.53	N/A	2.02 ± 2.29	N/A
*P*	<.01	<.01	N/A	<.01	N/A

CRP = C-reactive protein, SAA= serum amyloid A.

The number of patients with elevated/normal SAA and CRP levels during the non-inflammatory phase is shown in Table 4. CRP and SAA levels in the majority of patients with FMF were normal during the attack-free period, with only 12 patients (28.6%) having elevated acute-phase reactants. However, there were no significant differences in the markers that were elevated. In contrast, 10 (33.3%) patients with JIA had elevated acute-phase reactants. The relationship between CRP/SAA levels during the non-inflammatory phase and the clinical characteristics of patients with FMF and JIA is shown in Tables 5 and 6, respectively. The mean age of onset of JIA in patients with elevated acute-phase reactants was 3.0 (n = 10), and in those with normal acute-phase reactants was 6.1 (n = 20), respectively.

**Table 4:** The number of patients with SAA and CRP elevated/normal levels during non-inflammatory phase.

	FMF attack-free period (n = 42)	Postoperative appendicitis (n = 30)	JIA (n = 30)	Healthy controls (n = 20)
SAA elevated–CRP elevated	3 (7.1%)	1 (3.3%)	1 (3.3%)	0 (0%)
SAA normal–CRP elevated Attack-free/post op	5 (11.9%)	1 (3.3%)	0 (0%)	0 (0%)
SAA elevated–CRP normal Attack/pre op	4 (9.5%)	3 (10.0%)	9 (30.0%)	0 (0%)
SAA normal–CRP normal op	30 (71.4%)	25 (83.3%)	20 (66.7%)	20 (100.0%)

CRP = C-reactive protein, FMF = familial Mediterranean fever, JIA = juvenile idiopathic arthritis, SAA= serum amyloid A.

**Table 5:** Relationship between clinical characteristics of FMF patients and CRP/SAA levels during non-inflammatory phase.

FMF attack-free period (n = 42)		SAA elevated–CRP elevated	SAA normal–CRP elevated	SAA elevated–CRP normal	SAA normal–CRP normal	*P* *	*P* †	*P* ‡
All patients, n		3 (7.1%)	5 (11.9%)	4 (9.5%)	30 (71.4%)			
Gender, n						1.000	1.000	.602
	Male	1 (6.7%)	2 (13.3%)	2 (13.3%)	10 (66.7%)			
	Female	2 (7.4%)	3 (11.1%)	2 (7.4%)	20 (74.0%)			
Age of onset, mean		14.00	8.40	5.75	8.64 (n = 28)	.176	.885	.532
Diagnostic delay, mean (yr)		3.67	1.20	6.25	6.54 (n = 28)	.876	.174	.816
Arthritis, n		1 (11.1%)	1 (11.1%)	1 (11.1%)	6 (66.7%)	.523	1.000	1.000
Erysipelas-like rash, n		0 (0%)	0 (0%)	1 (50.0%)	1 (50.0%)	1.000	1.000	.225
Protracted febrile myalgia, n		0 (0%)	0 (0%)	0 (0%)	1 (100.0%)	1.000	1.000	1.000
MEFV mutation, n						.949	.744	.224
	M694V homozygous	2 (7.4%)	4 (14.8%)	2 (7.4%)	19 (70.4%)			
	Other	1 (7.7%)	1 (7.7%)	1 (7.7%)	10 (76.9%)			
	Unspecified	0 (0%)	0 (0%)	1 (50.0%)	1 (50.0%)			
Colchicine dose, mean		1.83	1.80	1.38	1.60 (n = 26)	.806	.913	.065
Biologic agent use, n (adalimumab, anakinra, canakinumab)		1 (11.1%)	0 (0%)	1 (11.1%)	7 (77.8%)	1.000	.559	1.000

CRP = C-reactive protein, FMF = familial Mediterranean fever, MEFV = Mediterranean fever gene, SAA= serum amyloid A.

* SAA elevated–CRP elevated vs SAA normal–CRP normal.

† SAA normal–CRP elevated vs SAA normal–CRP normal.

‡ SAA elevated–CRP normal vs SAA normal–CRP normal.

**Table 6:** Relationship between clinical characteristics of JIA patients and CRP/SAA levels during non-inflammatory phase.

JIA attack-free period (n = 30)		SAA elevated–CRP elevated	SAA normal–CRP elevated	SAA elevated–CRP normal	SAA normal–CRP normal	*P* *	*P* †	*P* ‡
All patients, n		1 (3.3%)	0 (0%)	9 (30.0%)	20 (66.7%)			
Gender, n						1.000	–	1.000
	Male	0 (0%)	0 (0%)	4 (28.6%)	10 (71.4%)			
	Female	1 (6.3%)	0 (0%)	5 (31.3%)	10 (62.5%)			
Age of onset, mean		2	-	3.11	6.1	.497	–	.160
Diagnostic delay, mean (yr)		1.00	-	0.67	0.50	.298	–	.572
JIA subgroup, n						.924	–	.836
	Oligoarticular	1 (8.3%)	0 (0%)	3 (25.0%)	8 (66.7%)			
	Polyarticular	0 (0%)	0 (0%)	5 (38.5%)	8 (61.5%)			
	Systemic	0 (0%)	0 (0%)	1 (50.0%)	1 (50.0%)			
	Psoriatic	0 (0%)	0 (0%)	0 (0%)	1 (100.0%)			
	Juvenile SpA	0 (0%)	0 (0%)	0 (0%)	1 (100.0%)			
	Unspecified	0 (0%)	0 (0%)	0 (0%)	1 (100.0%)			
Accompanying FMF, n		0 (0%)	0 (0%)	2 (33.3%)	4 (66.7%)	1.000	–	1.000
Small joint involvement, n		0 (0%)	0 (0%)	3 (42.9%)	4 (57.1%)	1.000	–	.642
Hip involvement, n		0 (0%)	0 (0%)	2 (50.0%)	2 (50.0%)	1.000	–	.568
Corticosteroid use, n		1 (5.6%)	0 (0%)	7 (38.9%)	10 (55.6%)	1.000	–	.234
Biologic agent use, n		0 (0%)	0 (0%)	6 (30.0%)	14 (70.0%)	.333	–	1.000

CRP = C-reactive protein, FMF = familial Mediterranean fever, JIA = juvenile idiopathic arthritis, SAA= serum amyloid A, SpA = spondyloarthritis.

* SAA elevated–CRP elevated vs SAA normal–CRP normal.

† SAA normal–CRP elevated vs SAA normal–CRP normal.

‡ SAA elevated–CRP normal vs SAA normal–CRP normal.

## 4. Discussion

This study showed that both CRP and SAA are sensitive to different inflammatory conditions, such as FMF, JIA, sepsis, and appendicitis. The resolution of inflammation, whether it is the postoperative status of the patient or the patient treated with anti-inflammatory medication, is associated with normal SAA and CRP levels. In the long-term follow-up of FMF, normal levels of both SAA and CRP reflect an attack-free period.

FMF requires close clinical follow-up owing to its symptoms and its most prominent complication, amyloidosis.^[7]^ During follow-up, it is recommended that the inflammatory marker level be maintained at a maximum of twice the upper normal limit.^[8]^ SAA was shown to be the most sensitive marker for disease screening and follow-up.^[8]^ Yalcinkaya et al have shown that in acute infections, SAA and CRP both increase, and the increase in SAA is more sensitive than the increase in CRP, yet still correlated.^[11]^ SAA has also been shown to reflect subclinical inflammation in patients with FMF.

In a study by Lachmann et al, 43 patients with FMF underwent serial measurements of both SAA and CRP during attacks and attack-free episodes.^[12]^ During attack-free episodes, the median SAA value was 2.2 mg/L, and the median CRP value was 1.3 mg/L. These normal values were elevated during clinical attacks (median SAA = 693 mg/L, median CRP = 115 mg/L). These acute-phase reactants were significantly higher in patients with a homozygous exon 10 mutation than in those with heterozygous exon 10 mutations. In addition, in a study by Lofty et al, both SAA and CRP levels were high during attack-free periods.^[13]^ Therefore, previously published data also suggest the non-inferiority of CRP to SAA.^[12-14]^

In our study, similar to the literature, SAA was substantially elevated in infectious and septic conditions (40–60 times higher), and CRP was also elevated to a lesser extent (20–35 times). Our data suggest that in assessing the inflammatory response, CRP and SAA are not different in terms of sensitivity.

SAA has been proposed to be used in FMF disease activity measurement because it plays a key role in the pathological accumulation of amyloid. However, clinical data are inadequate to demonstrate its effectiveness over other markers.^[15]^ The main objective of our study was to compare the sensitivities and specificities of SAA and CRP. The correlation between the 2 markers in different patient groups with inflammatory conditions was highlighted. This increased acute-phase response is observed in infectious conditions, such as sepsis and appendicitis, and autoinflammatory conditions, such as FMF and JIA. On the other hand, the almost equally normalized acute-phase response of CRP and SAA in JIA, acute appendicitis, and FMF patients when the inflammatory phase has resolved strengthens the proof of the non-inferiority of CRP to SAA.

C-reactive protein (CRP) is the universally accepted acute-phase reactant in clinical practice. It is used for a variety of clinical conditions, including FMF. In addition, CRP is known to play a role in subclinical inflammation during attack-free periods. In our study, we found that SAA correlated with other inflammatory conditions, including sepsis and acute appendicitis. Therefore, the specificity of SAA for autoinflammatory conditions is questionable and should be further tested in studies with longer follow-up durations. With equivocal data thus far, SAA should not replace CRP in clinical practice.

Our study has certain limitations. First, our methodology may not be sufficient when studying chronic diseases, as we may have overlooked the long-term complications of FMF. However, all our patients were treated with daily prophylactic colchicine, which hinders the development of the only known long-term complication of the disease.^[16]^ We do not have data on sepsis patients with resolved infections, but the normalization of the SAA and acute-phase reactants of appendicitis patients compensates for this limitation.

In conclusion, we did not observe any difference between CRP and SAA in terms of acute inflammatory response and normalization of the marker levels when the inflammation resolves. Because CRP has the benefit of accessibility, it seems to be more applicable to clinical practice. Further data from long-term follow-up will help us understand the role of these markers in the chronic management of the disease.

## Author contributions

**Conceptualization:** Oguzhan Selvi, Huri Ozdogan, Serdal Ugurlu.

**Data curation:** Oguzhan Selvi, Kerem Parlar, Mustafa Akker, Osman Simsek, Ozgur Kasapcopu.

**Formal analysis:** Bugra Han Egeli.

**Investigation:** Oguzhan Selvi, Kerem Parlar, Ibrahim Murat Bolayirli.

**Methodology:** Oguzhan Selvi.

**Project administration:** Sezgin Sahin.

**Supervision:** Sezgin Sahin, Osman Simsek, Ozgur Kasapcopur, Huri Ozdogan, Serdal Ugurlu.

**Writing – original draft:** Oguzhan Selvi, Kerem Parlar.

**Writing – review & editing:** Oguzhan Selvi, Bugra Han Egeli, Kerem Parlar, Serdal Ugurlu.
